# 

**Published:** 1902-12-15

**Authors:** 


					﻿THE
DENTAL REGISTER
EDITED BY
NELVILLE S. HOFF, D. D.S.,
ANN ARBOR, MICH.
Vol. LVI. DECEMBER 15, 1902.	No. 12.
PRICE, ONE DOLLAR A YEAR IN ADVANCE,
PUBLISHED MONTHLY BY
SAMUEL· A. CROCKER & CO.,
THE OHIO DENTAL AND SURGICAL DEPOT.
to :5S> NV. “tli st., Cinciliniiti, O.
Entered at the Postoffice at Cincinnati, 0., as second-class mail matter.
				

